# DGK α and ζ Activities Control T_H_1 and T_H_17 Cell Differentiation

**DOI:** 10.3389/fimmu.2019.03048

**Published:** 2020-01-15

**Authors:** Jialong Yang, Hong-Xia Wang, Jinhai Xie, Lei Li, Jinli Wang, Edwin C. K. Wan, Xiao-Ping Zhong

**Affiliations:** ^1^Division of Allergy and Immunology, Department of Pediatrics, Duke University Medical Center, Durham, NC, United States; ^2^Department of Microbiology, Immunology, and Cell Biology, West Virginia University School of Medicine, Morgantown, WV, United States; ^3^Department of Neuroscience, West Virginia University School of Medicine, Morgantown, WV, United States; ^4^Department of Immunology, Duke University Medical Center, Durham, NC, United States; ^5^Hematologic Malignancies and Cellular Therapies Program, Duke Cancer Institute, Duke University Medical Center, Durham, NC, United States

**Keywords:** Th differentiation, Th17, Th1, mTOR, DGK, airway inflammation

## Abstract

CD4^+^ T helper (T_H_) cells are critical for protective adaptive immunity against pathogens, and they also contribute to the pathogenesis of autoimmune diseases. How T_H_ differentiation is regulated by the TCR's downstream signaling is still poorly understood. We describe here that diacylglycerol kinases (DGKs), which are enzymes that convert diacylglycerol (DAG) to phosphatidic acid, exert differential effects on T_H_ cell differentiation in a DGK dosage-dependent manner. A deficiency of either DGKα or ζ selectively impaired T_H_1 differentiation without obviously affecting T_H_2 and T_H_17 differentiation. However, simultaneous ablation of both DGKα and ζ promoted T_H_1 and T_H_17 differentiation *in vitro* and *in vivo*, leading to exacerbated airway inflammation. Furthermore, we demonstrate that dysregulation of T_H_17 differentiation of DGKα and ζ double-deficient CD4^+^ T cells was, at least in part, caused by increased mTOR complex 1/S6K1 signaling.

## Introduction

CD4^+^ T helper (T_H_) cells play a central role in orchestrating adaptive immune response to pathogens and also contribute to autoimmune diseases (1, 2). After antigen stimulation, naïve CD4^+^ T cells differentiate into discrete subsets of effector T_H_ cells with distinct functions and cytokine profiles. Interferon-γ (IFN-γ)-producing T_H_1 cells, induced by IL-12 and directed by transcriptional factor T-bet, are critical for the clearance of intracellular pathogens (3, 4). T_H_2 cells, which secrete IL-4, IL-5, and IL-13 and are controlled by GATA-3, are crucial for protection against parasites and extracellular pathogens (5, 6). T_H_17 cells produce IL-17A, IL-17F, and IL-22, and play an important role in the control of specific pathogens such as fungi. T_H_17 differentiation is driven by a combination of TGF-β and IL-6 and requires transcriptional factor RORγt and RORα. IL-23 promotes T_H_17 responses by enhancing their survival and stabilization (7–12).

Despite their importance in host immunity against pathogens, T_H_ cells can be pathogenic and contribute to various diseases. Both exaggerated and defective T_H_1 response has been linked to the induction of autoimmune diseases (13–15). T_H_2 cells contribute to allergies and asthma (16, 17). T_H_17 cells are associated with many autoimmune and inflammatory diseases such as psoriasis, inflammatory bowel diseases, rheumatoid arthritis, type 1 diabetes, and multiple sclerosis (8, 11, 18–20). Thus, understanding how T_H_ responses are regulated is important to manipulate immune responses, to improve host defense against microbial infection, and to treat autoimmune diseases.

Engagement of the TCR on naïve CD4^+^ T cells is essential for their activation and further differentiation to T_H_ cells (21, 22). Evidence has revealed that TCR signal strength and downstream signaling pathways as well as cytokine and costimulatory signals shape T_H_ lineage differentiation (23–26). A critical event after TCR engagement is the generation of the second messenger diacylglycerol (DAG) by activated PLCγ1. DAG associates with and allosterically activates RasGRP1 and PKCθ, leading to the activation of the Ras-Erk1/2-AP1 and PKCθ-IKK-NFκB signaling pathways, respectively, and is indispensable for T cell activation (27–30). Since it has been demonstrated that both Ras- and PKCθ-mediated signal cascades are involved in T_H_ differentiation (31–34), it is important to investigate if DAG concentrations should be tightly controlled during T_H_ differentiation.

DAG kinases (DGKs), a family of enzymes that catalyze phosphorylation of DAG to generate phosphatidic acid (PA), are employed to inhibit DAG-mediated signaling following TCR engagement in both thymocytes and peripheral T cells (28–30). DGKα and ζ, isoforms that express at high levels in T cells, have been demonstrated to inhibit the activation of both Ras-Erk and PKCθ-NFκB cascades as well as mTOR signaling (35–37). They regulate conventional αβT cell, iNKT cell, mucosal associated invariant T cell, and regulatory T cell development, negatively control T cell activation, regulate CD8 T cell mediated anti-viral responses and activation induced T cell death, promote T cell anergy, and inhibit anti-tumor responses (27, 38–55). However, the role of DGKs in T_H_ differentiation is unknown. We report here that a deficiency of either DGKα or ζ selectively impairs T_H_1 cell differentiation, but the loss of both DGK isoforms enhances CD4^+^ naïve T cells differentiating into T_H_1 and T_H_17 *in vitro* and *in vivo*, establishing DGK activity as a critical regulator of effector CD4^+^ T cell differentiation.

## Materials and Methods

### Mice

DGKα^−/−^, DGKζ^−/−^, and ERCre mice were generated as previously described (38, 39, 56). DGKζ^*f*/*f*^ mice were generated by introducing two LoxP sites that flank exons 10–14 of the *Dgkz* locus (57). TCR transgenic OT2 mice were purchased from the Jackson Laboratory and were cross-bred with DGKα^−/−^ζ^*f*/*f*^ ERCre mice to generate DGKα^−/−^ζ^*f*/*f*^ OT2 ERCre mice in specific pathogen-free facilities at Duke University Medical Center. The experiments in this study were performed according to a protocol approved by the Institutional Animal Care and Usage Committee of Duke University. DGKα^−/−^ζ^*f*/*f*^ or DGKα^−/−^ζ^*f*/*f*^ OT2 ERCre mice were intraperitoneally injected with tamoxifen (100 mg/kg body weight) on the first, second, and fifth day to delete DGKζ, and mice were then euthanized for experiments on the eighth day.

### Reagents and Antibodies

Iscove's modified Dulbecco's medium (IMDM) was supplemented with 10% (vol/vol) FBS, penicillin/streptomycin, and 50 μM 2-mercaptoethanol (IMDM-10). Fluorescence-conjugated anti-mouse antibodies CD4 (GK1.5), TCRVα2 (B20.1), CD44 (IM7), CD62L (MEL-14), Thy1.1 (OX-7), Thy1.2 (58-2.1), T-bet (4B10), IFN-γ (XMG1.2), IL-4 (11B11), IL-17A (TC11-18H10.1), and IL-17F (9D3.1C8) were purchased from BioLegend; anti-mouse antibodies for RORγt (AFKJS-9) and Foxp3 (FJK-16s) were purchased from eBioscience. Cell death was determined by Live/Dead Fixable Violet Dead Cell Stain (Invitrogen).

### Flow Cytometry

Standard protocols were used to prepare single cell suspensions from the spleen and lymph nodes of mice (in IMDM containing 10% FBS and antibiotics). Red blood cells were lysed using an ACK buffer. Samples were subsequently stained with antibodies in PBS containing 2% FBS and collected on a BD FACSCanto II cytometer. Intracellular staining for T-bet and RORγt was performed using the eBioscience Foxp3 Staining Buffer Set. Intracellular staining for IFNγ, IL-4, IL-17A, and IL-17F was performed using the BD Biosciences Cytofix/Cytoperm and Perm/Wash solutions.

### *In vitro* T_**H**_ Differentiation

CD4^+^ T cells were purified from the spleen and LN with anti-CD4 microbeads (Miltenyi Biotec) and then were further sorted as naïve CD4^+^CD62L^hi^CD44^lo^CD25^−^. Sorted cells were activated with plate-bound anti-CD3 (5 μg/ml, 1452C11, Bio Xcell) and soluble anti-CD28 (1 μg/ml, PV1, BioXcell) for 4–5 days with various combinations of cytokines and antibodies. For the non-polarizing (T_H_0) condition, naïve cells were cultured in the presence of hIL-2 (100 U/ml, Peprotech). For the T_H_1 condition, naïve cells were cultured with hIL-2 (100 U/ml), mIL-12 (20 ng/ml, Peprotech), and anti-mIL4 (10 μg/ml, 11B11, Bio Xcell) for 4 days. For the T_H_2 condition, naïve cells were polarized in the presence of hIL-2 (100 U/ml), mIL-4 (20 ng/ml, Peprotech), and anti-IFNγ (10 μg/ml, XMG1.2, BioXcell) for 5 days. For the T_H_17 condition, naïve cells were cultured with hTGF-β1 (5 ng/ml, Peprotech), mIL-6 (25 ng/ml, Peprotech), anti-mIL4 (10 μg/ml), and anti-IFNγ (10 μg/ml) for 4 days. For iTreg induction, 100 U/ml of hIL-2 and 1 ng/ml TGFβ (Peprotech) were included in the culture for 4 days, followed by intracellular Foxp3 staining. To assess proliferation, sorted naïve CD4^+^ T cells were labeled with CellTrace™ Violet (CTV, ThermoFisher) before cultured in different polarization conditions. For the inhibition assay, 10 μM S6K inhibitor (PF-4708671, Sigma) and 1 nM rapamycin were added to the T_H_1 and T_H_17 polarizing conditions at the beginning of culture, and cells were cultured for 4 days. At the end of polarizing, cells were stimulated with PMA (50 ng/ml) and ionomycin (500 ng/ml) in the presence of GolgiPlug (1 ng/ml) for 4–5 h. This was followed by cell surface and intracellular staining for appropriated cytokines.

### Adoptive Transfer, Immunization, and Airway Inflammation

TCRVα2^+^ cells from splenocytes and LN cells for TCR OTII transgenic mice were enriched using MACS magnetic beads and Miltenyi Biotec LS columns. About 100 million cells in 500 μl of IMDM-10 were incubated with the PE-TCRVα2 antibody (1:100 dilution) and then with anti-PE magnetic beads to isolate TCRVα2^+^ cells according to the manufacturer's protocol. Enriched samples were stained with anti-CD4, −CD44, and −CD62L antibodies and sorted on a MoFlo Astrios sorter to obtain viable CD4^+^TCRVα2^+^CD44^−^CD62L^+^ naïve OT2 T cells. Naïve WT or DGKα^−/−^ζ^*f*/*f*^ OT2 cells (Thy1.1^−^Thy1.2^+^, 1.5 × 10^6^ cell/mouse) were intravenously injected into sex-matched recipients (Thy1.1^+^Thy1.2^+^). Recipient mice were immunized by subcutaneous injection in the inguinal region with 100 μg/mouse OVA_323−339_ peptide emulsified in the CFA 24 h after adoptive transfer and were euthanized to harvest the spleen and drain inguinal lymph nodes on the seventh day after immunization. Splenocytes and dLN cells were stimulated with PMA and ionomycin in the presence of GolgiPlug for 4–5 h or stimulated with 10 μg/ml OVA_323−339_ for 2 days in the presence of 1 ng/ml GolgiPlug in the last 5 h. Cell surface and intracellular staining for appropriated cytokines were subsequently performed.

For airway inflammation, OTII T cell recipient mice were intranasally injected with 25 μl of 2.5 mg/ml OVA_323−339_ peptide in PBS daily for 3 consecutive days starting 24 h after adoptive transfer. Mice were euthanized on the eighth day after adoptive transfer for collection of BALF. Lungs were fixed in 10% formalin and thin-sectioned for hematoxylin and eosin (H&E) staining. Spleen and draining mediastinal LNs were harvested for cytokine analysis.

### ELISA

Cultured supernatant or BALF samples were appropriately diluted and IFNγ, IL-4, and IL-17A concentrations were determined using Mouse ELISA max kits (BioLegend) according to the manufacturer's instructions.

### Real-Time RT-PCR

Cells were lysed in Trizol for RNA preparation. The first strand cDNA was made using the iScript Select cDNA Synthesis Kit (Biorad). Real-time quantitative PCR was conducted using Eppendorf realplex^2^. Expressed levels of target mRNAs were normalized with β-actin and calculated using the 2^−ΔΔ*CT*^ method. Primers used in this study are listed as following: DGKα Forward: GATGCAGGCACCCTGTACAAT, Reverse: GGACCCATAAGCATAGGCATCT; DGKζ Forward: CGGCTGCCTGGTGTAGACA, Reverse: GCACCTCCAGAGATCCTTGATG; IFN-γ Forward: GCGTCATTGAATCACACCTG, Reverse: TGAGCTCATTGAATGCTTGG; IL-4 Forward: ACAGGAGAAGGGACGCCA, Reverse: GAAGCCCTACAGACGAGCTCA; IL-17A Forward: GCTCCAGAAGGCCCTCAGA, Reverse: CTTTCCCTCCGCATTGACA; Tbx21 Forward: GGTGTCTGGGAAGCTGAGAG, Reverse: GAAGGACAGGAATGGGAACA; GATA-3 Forward: AACCACGTCCCGTCCTACTA, Reverse: AGAGATCCGTGCAGCAGA; RORc Forward: CGACTGGAGGACCTTCTACG, Reverse: TTGGCAAACTCCACCACATA; RORα Forward: CCATGCAAGATCTGTGGAGA, Reverse: CAGGAGTAGGTGGCATTGCT; β-actin Forward: TGTCCACCTTCCAGCAGATGT, Reverse: AGCTCAGTAACAGTCCGCCTAGA.

### Western Blot Analysis

*In vitro*-cultured T_H_ cells were lysed in lysis buffer (1% Nonidet P-40, 150 mM NaCl, 50 mM Tris, pH 7.4) with freshly added protease and phosphatase inhibitors. Samples were subjected to immunoblotting analysis, and probed with anti-pS6 (S235/236), -pErk1/2, -total S6, -total Erk1/2, and β-actin antibodies (Cell Signaling Technology).

### Statistical Analysis

Data are presented as mean ± SEM, and statistical significance was determined by two-tailed Student's *t*-test. The *p*-values are defined as follows: ^*^p < 0.05, ^**^p < 0.01, ^***^p < 0.001.

## Results

### Deficiency of Either DGKα or ζ Impaired T_**H**_1 Cell Differentiation

**Figure 1:**
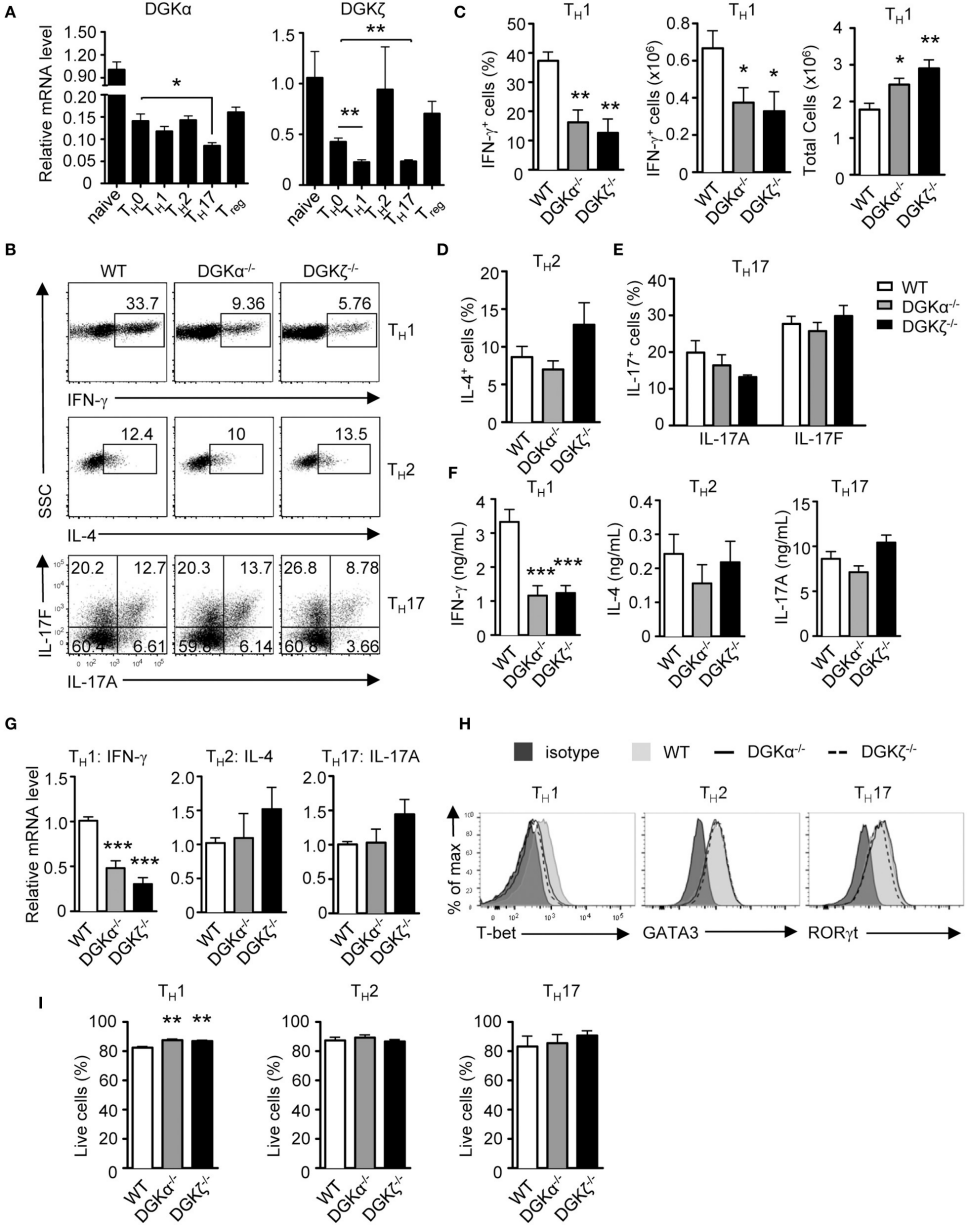
Effects of DGKα or DGKζ deficiency on T_H_ differentiation. **(A)** Relative mRNA expression level of DGKα and DGKζ in WT CD4^+^ T cells before and after 48-h culturing in the indicated T_H_ and iTreg differentiation conditions. Mean ± SEM of triplicates are shown and represent three experiments. **(B–H)** WT, DGKα^−/−^, and DGKζ^−/−^ naïve CD4^+^ T cells were cultured in T_H_1, T_H_2, and T_H_17 conditions *in vitro* for 4–5 days. **(B)** Representative dot plots of cytokine-producing cells gated on CD4^+^ T cells after PMA and ionomycin stimulation for 4–5 h. **(C)** Bar graphs show mean ± SEM of percentages and numbers of IFNγ^+^ cells and total CD4^+^ T cells. **(D)** Bar graphs show mean ± SEM of percentages IL-4^+^ cells. **(E)** Bar graphs show mean ± SEM of IL-17A^+^ and IL-17F^+^ cells. **(F)** Cytokine concentrations in culture supernatants collected at 96 h. **(G)** Relative mRNA levels of cytokines in indicated T_H_ conditions after 48 h of incubation. **(H)** Overlaid histograms of intracellular staining of indicated transcription factors under indicated T_H_ conditions for 60 h. **(I)** Bar graphs show mean ± SEM of survival rates of CD4^+^ T cells under different T_H_ conditions. Data shown are representative of or pooled from at least three independent experiments. *P < 0.05; **P < 0.01; ***P < 0.001 (Student *t*-test).

DGKα and ζ are dynamically regulated during T cell development and activation (27, 35, 39, 40). We found that DGKα mRNA was decreased in T_H_0, T_H_1, T_H_2, T_H_17, and iTregs compared with naïve CD4^+^ T cells. DGKζ mRNA also was decreased in T_H_0, T_H_1, and T_H_17 cells but not in T_H_2 and iTregs compared with naïve CD4^+^ T cells (Figure 1A). Both DGKα and ζ appeared more significantly down-regulated in T_H_1 and T_H_17 conditions than in T_H_0 condition. To examine the role of DGKα and ζ in T_H_ differentiation, WT, DGKα^−/−^, and DGKζ^−/−^ CD44^−^CD62L^+^ naïve CD4^+^ T cells were cultured in T_H_1, T_H_2, and T_H_17 polarization conditions *in vitro* for 4–5 days. DGKα^−/−^ or DGKζ^−/−^ CD4^+^ T cells displayed impaired differentiation to T_H_1 cells, which was indicated by decreases of IFN-γ^+^ cells in both percentages and numbers (Figures 1B,C), IFN-γ concentration in culture supernatants (Figure 1F), and IFN-γ mRNA levels (Figure 1G), accompanying the decreased expression of T-bet (Figure 1H). However, total CD4^+^ T cells numbers were increased in the absence of either DGKα or ζ during T_H_1 polarization (Figure 1C), suggesting that impaired T_H_1 differentiation of DGKα^−/−^ or DGKζ^−/−^ CD4^+^ T cells did not result from decreased expansion. In contrast, T_H_2 and T_H_17 differentiation was not obviously affected by DGKα or ζ deficiency. This was reflected by similar percentages of IL-4^+^ or IL-17^+^ cells (Figures 1B,D,E) and similar levels of IL-4 or IL-17A proteins in culture supernatants (Figure 1F) and mRNAs (Figure 1G), which correlated with comparable expression of GATA-3 or RORγt (Figure 1H). Both DGKα^−/−^ CD4^+^ T cells and DGKζ^−/−^ CD4^+^ T cells displayed slightly improved survival under the T_H_1 condition and had similar survival rates under T_H_2 and T_H_17 conditions (Figure 1I), suggesting that their reduced T_H_1 responses were not due increased cell death. Together, these data suggested individual DGKα and DGKζ are required for T_H_1 differentiation, but are dispensable for T_H_2 and T_H_17 development *in vitro*.

### Deficiency of Both DGKα and ζ Promoted T_**H**_1 and T_**H**_17 Differentiation

**Figure 2:**
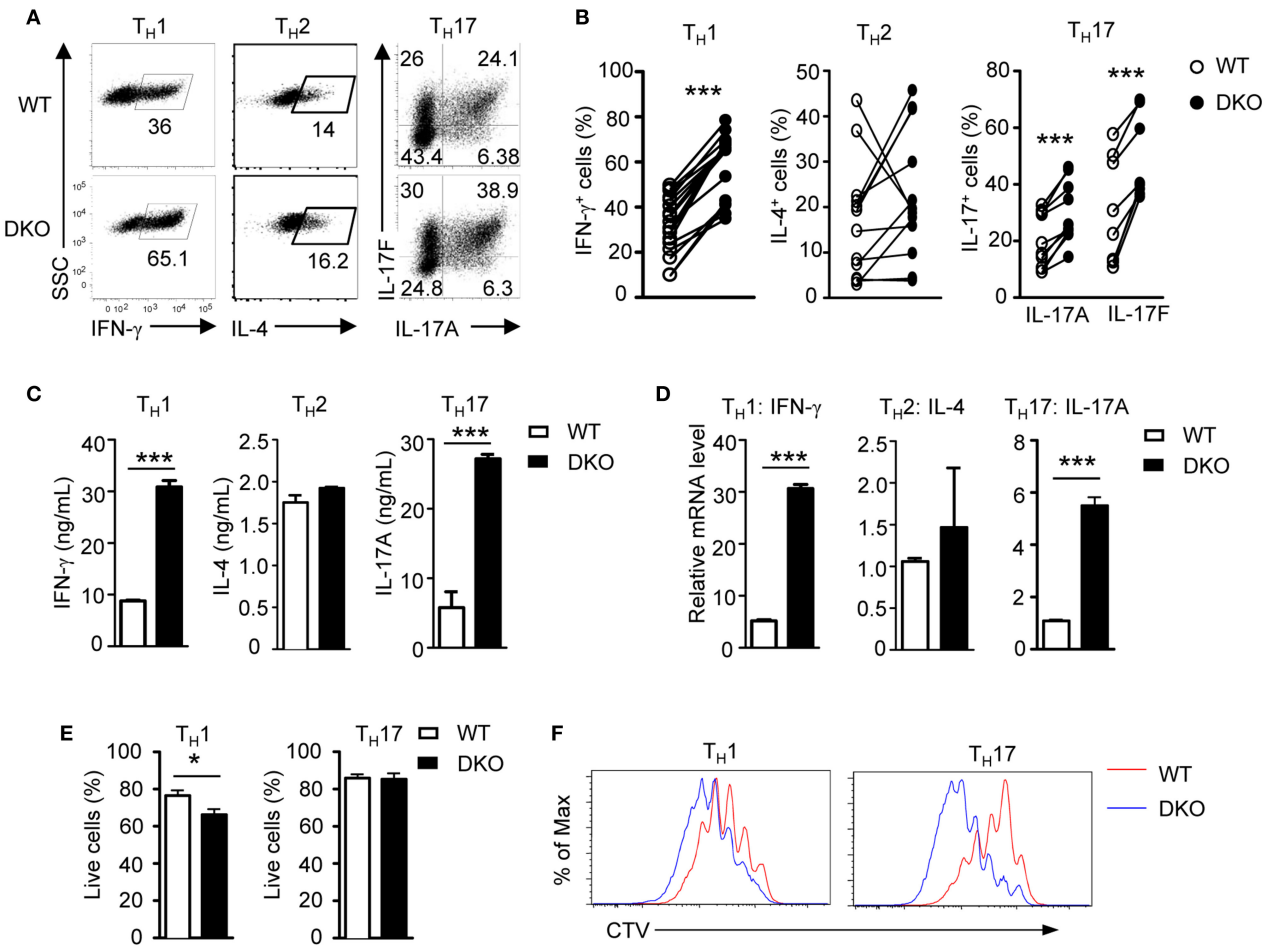
A deficiency of both DGKα and ζ promotes T_H_1 and T_H_17 differentiation *in vitro*. WT and DKO naïve CD4^+^ T cells were similarly cultured in T_H_ polarization conditions and analyzed as shown in Figure 1. **(A)** Representative dot plots of cytokine-producing cells gated on CD4^+^ T cells. **(B)** Percentages of cytokine-producing cells in indicated T_H_ conditions. **(C)** Cytokine concentrations in culture supernatants. **(D)** Relative mRNA levels in indicated T_H_ cells. **(E)** Survival rates of CD4^+^ T cells under T_H_1 and T_H_17 conditions. **(F)** Representative histograms showing CD4^+^ T cell proliferation under T_H_1 and T_H_17 conditions using a CTV dilution assay. Data shown represent or are pooled from at least eight **(A,B)** or four **(C,D)** independent experiments. *P < 0.05; ***P < 0.001 as determined by a paired Student *t*-test.

DGKα and ζ promote T cell and iNKT cell maturation synergistically in the thymus (52, 54). To determine if DGKα and ζ exert a synergistic role during T_H_ differentiation, we generated DGKα^−/−^ζ^*f*/*f*^-ERCre (DKO) mice so that both DGKα and ζ were ablated after tamoxifen-induced deletion of DGKζ. In contrast to DGKα or ζ single-knockout T cells, DKO CD4^+^ naïve T cells showed enhanced capacity to differentiate into both T_H_1 and T_H_17 cells but similar T_H_2 differentiation compared with their WT counterparts (Figures 2A,B), coinciding with increased IFN-γ and IL-17A but not IL-4 concentration in culture supernatants (Figure 2C) and IFN-γ and IL-17A mRNA levels in these cells (Figure 2D). DKO CD4^+^ T cells displayed slightly decreased survival rate under T_H_1 but similar survival rate under T_H_17 polarization conditions, suggesting that their enhanced T_H_1 and T_H_17 responses were not due to improved survival (Figure 2E). However, under both T_H_1 and T_H_17 conditions, DKO CD4^+^ T cells proliferated more vigorously than WT controls, which might contribute to their enhanced T_H_1 and T_H_17 responses (Figure 2F). In contrast to T_H_1 and T_H_17 differentiation, iTreg cell induction was not obviously different between WT and DKO naïve CD4^+^ T cells (WT iTreg percentages: 62.49 ± 9.186 *n* = 7; DKO iTreg percentages: 53.84 ± 8.465 *n* = 7; *P* = 0.5022). Together, these results indicated that deficiency of both DGKα and ζ promoted T_H_1 and T_H_17 differentiation with minimal effects on T_H_2 or iTreg cell differentiation *in vitro*.

### Loss of Both DGKα and ζ Prompted T_**H**_1 and T_**H**_17 Differentiation *in vivo*

**Figure 3:**
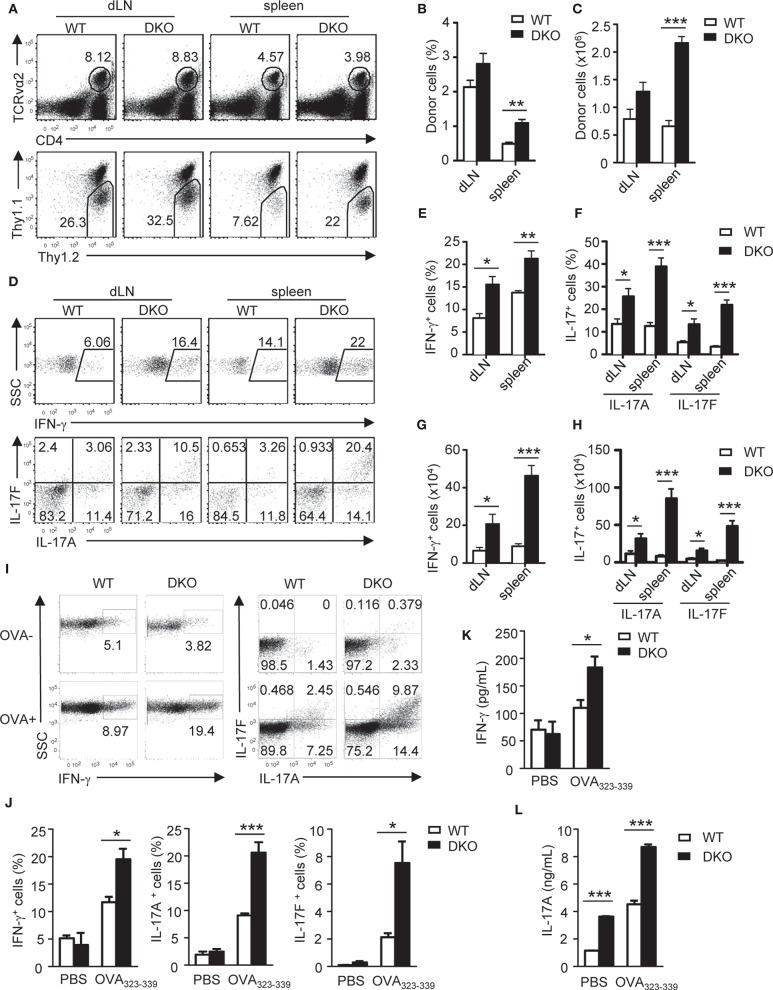
Loss of both DGKα and ζ enhances T_H_1 and T_H_17 differentiation *in vivo*. Thy1.1^+^Thy1.2^+^ congenic mice *in vivo* injected with 1.5 × 10^6^ Thy1.1^−^Thy1.2^+^TCRVα2^+^CD4^+^ WT or DKO naïve OT2 T cells on day −1 were immunized with OVA_323−339_ peptide in CFA on day 0. Spleens and dLNs were harvested on the seventh day after immunization. **(A)** Representative dot plots of dLN cells and splenocytes. Top panels: CD4 and TCRVα2 staining. Bottom panels: Thy1.1 vs. Thy1.2 staining of the gated TCRVα2^+^CD4^+^ population. **(B,C)** Mean ± SEM of percentages **(B)** and number **(C)** of donor-derived OT2 T cells in dLNs and spleens (*n* = 4). **(D–H)** dLN cells and splenocytes were stimulated with PMA and ionomycin for 4–5 h in the presence of GolgiPlug, followed by cell surface and intracellular staining. **(D)** Representative dot plots of indicated cytokines in gated donor-derived OT2 cells. **(E–H)** Mean ± SEM of percentages of IFN-γ-producing cells **(E)** and IL-17-producing cells **(F)** as well as total numbers of donor-derived IFN-γ-producing **(G)** and IL17-producing **(H)** OT2 T cells. **(I–K)** Splenocytes and dLN cells were stimulated with (OVA+) or without (OVA–) OVA_323−339_ for 2 days, with the addition of GolgiPlug in the last 5 h, and then were cell surfaced and intracellular stained for OT2 T cells and cytokine expression. **(I)** Representative dot plots of indicated ctyokine-producing cells in gated donor-derived OT2 cells. **(J)** Percentages of donor-derived cytokine-producing OT2 T cells (*n* = 4). **(K,L)** IFN-γ **(K)** and IL-17A **(L)** concentrations in culture supernatant harvested before adding GolgiPlug (*n* = 3). Data shown are representative of two independent experiments. *P < 0.05; **P < 0.01; ***P < 0.001 as determined by the Student *t*-test.

To further determine the impact of DGKα and ζ double deficiency on T_H_ differentiation *in vivo*, we generated DKO mice carrying the OT2 TCR transgene, which recognizes chicken ovalbumin peptide 323-339 (OVA_323−339_) in the context of I-A^b^ (58) and adoptively transferred WT- or DKO-naïve OT2 T cells (Thy1.1^−^Thy1.2^+^CD4^+^TCRVα2^+^) into congenic Thy1.1^+^Thy1.2^+^ recipients. Recipient mice were immunized with OVA_323−339_ peptide emulsified in complete Freund's adjuvant (CFA) 1 day after the transfer. Seven days after immunization, donor-derived DKO OT2 T cells were increased in both percentages and numbers in the spleen and draining lymph nodes (dLNs) compared with WT controls (Figures 3A–C). In addition, higher percentages of DKO OT2 T cells expressed IFN-γ, IL-17A, and IL-17F than WT controls following *in vitro* PMA and ionomycin stimulation for 4 h (Figures 3D–F). Because of increased DKO OT2 T cell numbers, DKO OT2 T_H_1 and T_H_17 cell numbers were much greater than WT controls in dLNs and particularly in the spleen (Figures 3G,H). Moreover, DKO OT2 T cells contained more IFN-γ-, IL-17A-, and IL-17F-positive cells, which was detected by intracellular staining (Figures 3I,J), and secreted more cytokines to culture supernatants, which was detected by ELISA (Figures 3K,L), than their WT controls following stimulation with OVA_323−339_ peptide for 2 days. Together, these results demonstrated that the deficiency of both DGKα- and ζ-enhanced T_H_1 and T_H_17 polarization and expansion *in vivo* via cell intrinsic mechanisms.

### Accumulation of T_**H**_1 and T_**H**_17 Cells in the Absence of DGKα and ζ Caused Severe Airway Inflammation

**Figure 4:**
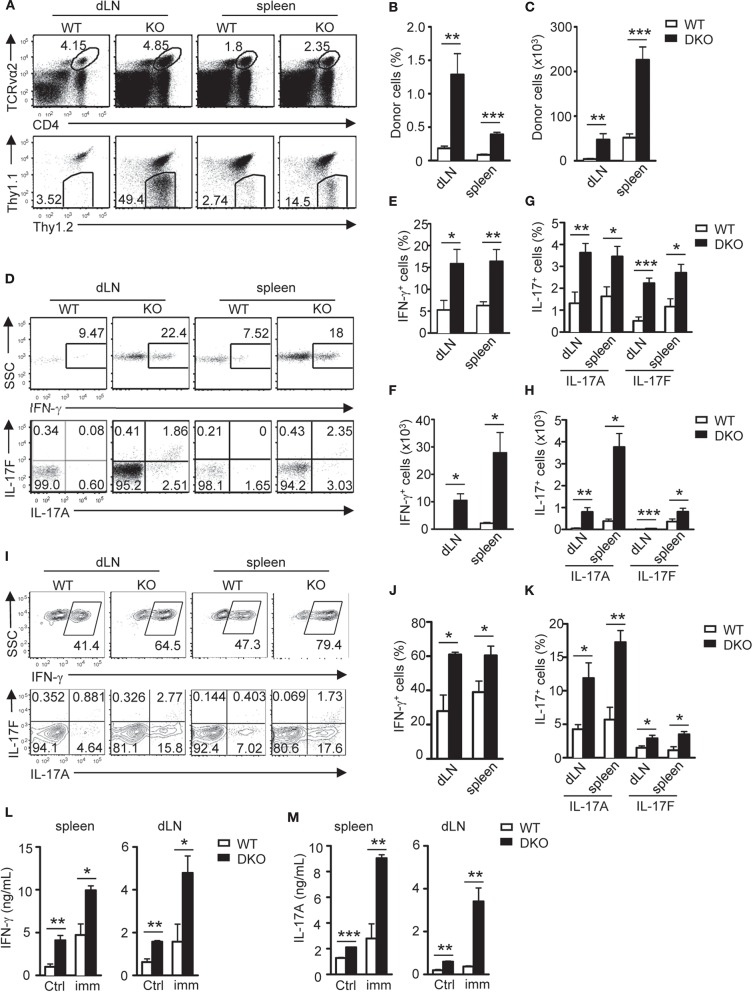
DGKαζDKO-enhanced airway Th17 responses. Thy1.1^+^Thy1.2^+^ congenic mice injected with 1.5 × 10^6^ Thy1.1^−^Thy1.2^+^Vα2^+^CD4^+^ WT or DKO naïve OT2 T cells on day −1 were intranasally injected with OVA_323−339_ peptide on days 0, 1, and 2. Draining mediastinal lymph nodes and spleens were harvested on the seventh day. **(A)** Representative dot plots of dLN cells and splenocytes. Top panels: CD4 vs. TCRVα2 staining. Bottom panels: Thy1.1 vs. Thy1.2 staining of the gated TCRVα2^+^CD4^+^ population. **(B,C)** Percentages **(B)** and number **(C)** of donor-derived OT2 T cells in dLNs and spleens. **(D–H)** Splenocytes and dLN cells from recipients were stimulated with PMA and ionomycin for 4–5 h, followed by cell surface and intracellular staining. **(D)** Representative dot plots of indicated cytokines in donor-derived OT2 T cells. **(E,F)** Percentages **(E)** and number **(F)** of donor-derived IFN-γ-producing OT2 T cells. **(G,H)** Percentages **(G)** and number **(H)** of donor-derived IL-17A- and IL-17F-producing OT2 T cells. **(I–M)** Splenocytes and dLN cells were stimulated with OVA_323−339_ for 2 days with GolgiPlug added in the last 5 h, followed by cell surface and intracellular staining. **(I)** Representative dot plots of indicated cytokine staining in gated donor-derived OT2 T cells. **(J,K)** Percentages of IFN-γ- **(J)** and IL-17-producing cells **(K)** in donor OT2 T cells. **(L,M)** IFN-γ **(L)** and IL-17A **(M)** concentrations in culture supernatants. Data shown are representative of or calculated from two independent experiments (*n* = 8). *P < 0.05; **P < 0.01; ***P < 0.001 as determined by the Student *t*-test.

**Figure 5:**
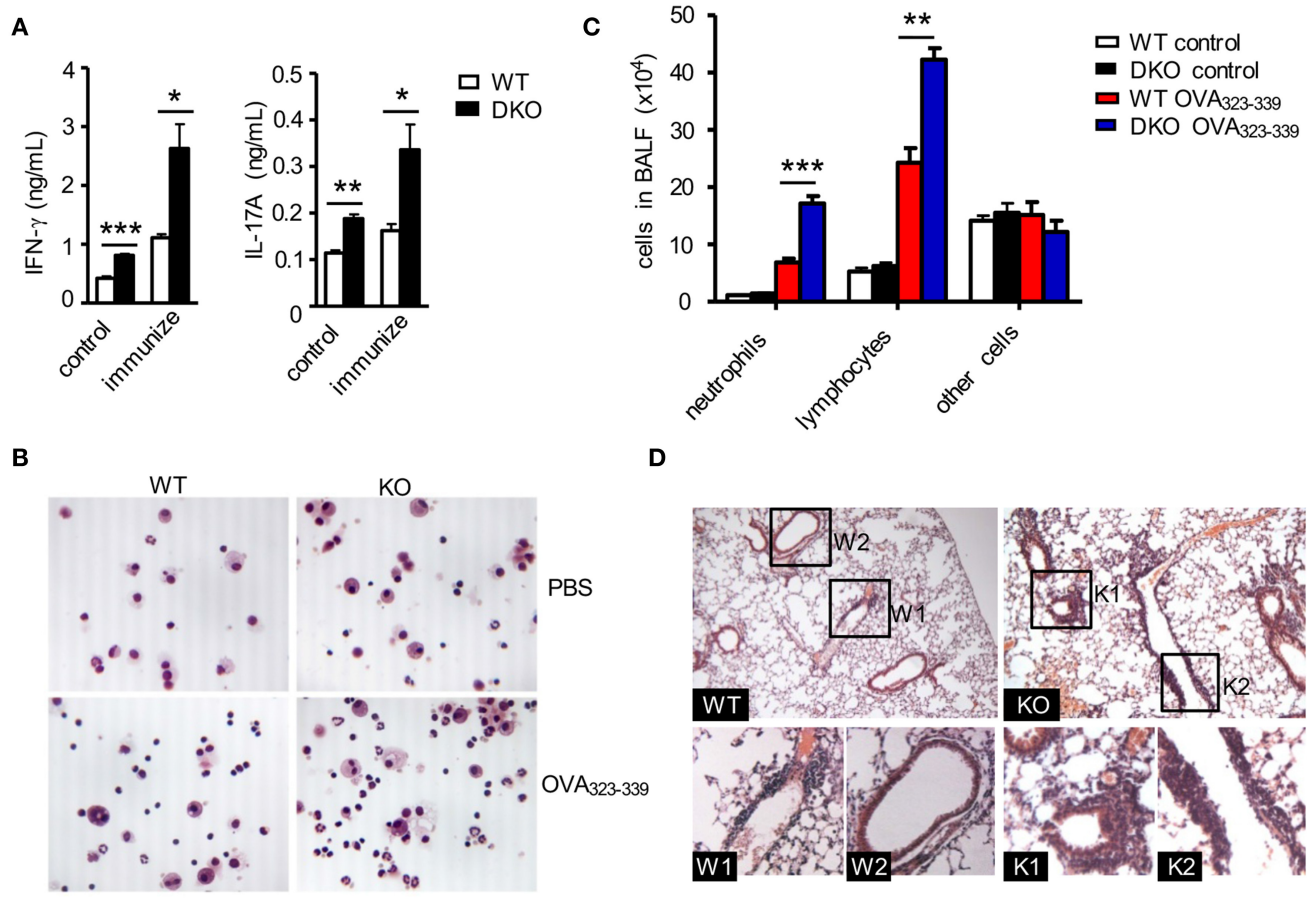
DGKαζDKO-exacerbated CD4^+^ T cell-mediated airway inflammation. The same WT and DKO OT2 T cell recipient mice intranasally injected with OVA in Figure 4 were utilized to collect BALF and lung tissues. **(A)** IFNγ and IL-17A concentrations in BALF. **(B)** Giemsa staining of fresh-harvested WT and DKO BALF from mice with or without OVA_323−339_ challenge. **(C)** Leukocyte differentials in BALF calculated based on Giemsa staining. **(D)** H&E staining of thin lung sections. Data shown are representative of two independent experiments. *P < 0.05; **P < 0.01; ***P < 0.001 as determined by the Student *t*-test.

T_H_17 cells promote airway inflammation and hyper-responsiveness via recruiting neutrophils and induce airway smooth muscle contraction, which contributes to the severe form of asthma (59, 60). To determine if dysregulated T_H_ responses of DKO CD4^+^ T cells impact airway inflammation, we adoptively transferred naïve WT and DKO OT2 cells (Thy1.2^+^) into WT Thy1.1^+^Thy1.2^+^ congenic mice on day −1 and then intranasally injected OVA_323−339_ peptide into the recipient mice on days 0, 1, and 2. On the seventh day, we detected at least four-fold more DKO OT2 cells in both percentages and numbers in the draining mediastinal lymph nodes and spleen in recipient mice than their WT counterparts (Figures 4A–C). DKO donor-derived OT2 cells in both dLNs and spleens produced more IL-17A and IL-17F as well as IFN-γ in response to *in vitro* stimulation with PMA and ionomycin for 4 h (Figures 4D–H) or with OVA_323−339_ peptide for 2 days (Figures 4I–M). Concordantly, both IFN-γ and IL-17A levels in bronchoalveolar lavage fluid (BALF) were elevated in recipients with DKO OT2 T cells compared with those with WT OT2 T cells (Figure 5A). Moreover, DKO OT2 cell recipients contained more neutrophils and lymphocytes than those with WT control in BALF (Figures 5B,C) and in interstitial lung tissues that surround the bronchioles (Figure 5D). Together, these results demonstrated that DGKα and ζ deficiencies in CD4^+^ T cells exacerbated airway inflammation, likely as a result of enhanced T_H_17 responses to protein allergens.

### Effects of DGKαζ Double Deficiency on Expression of Critical Lineage Transcription Factors

**Figure 6:**
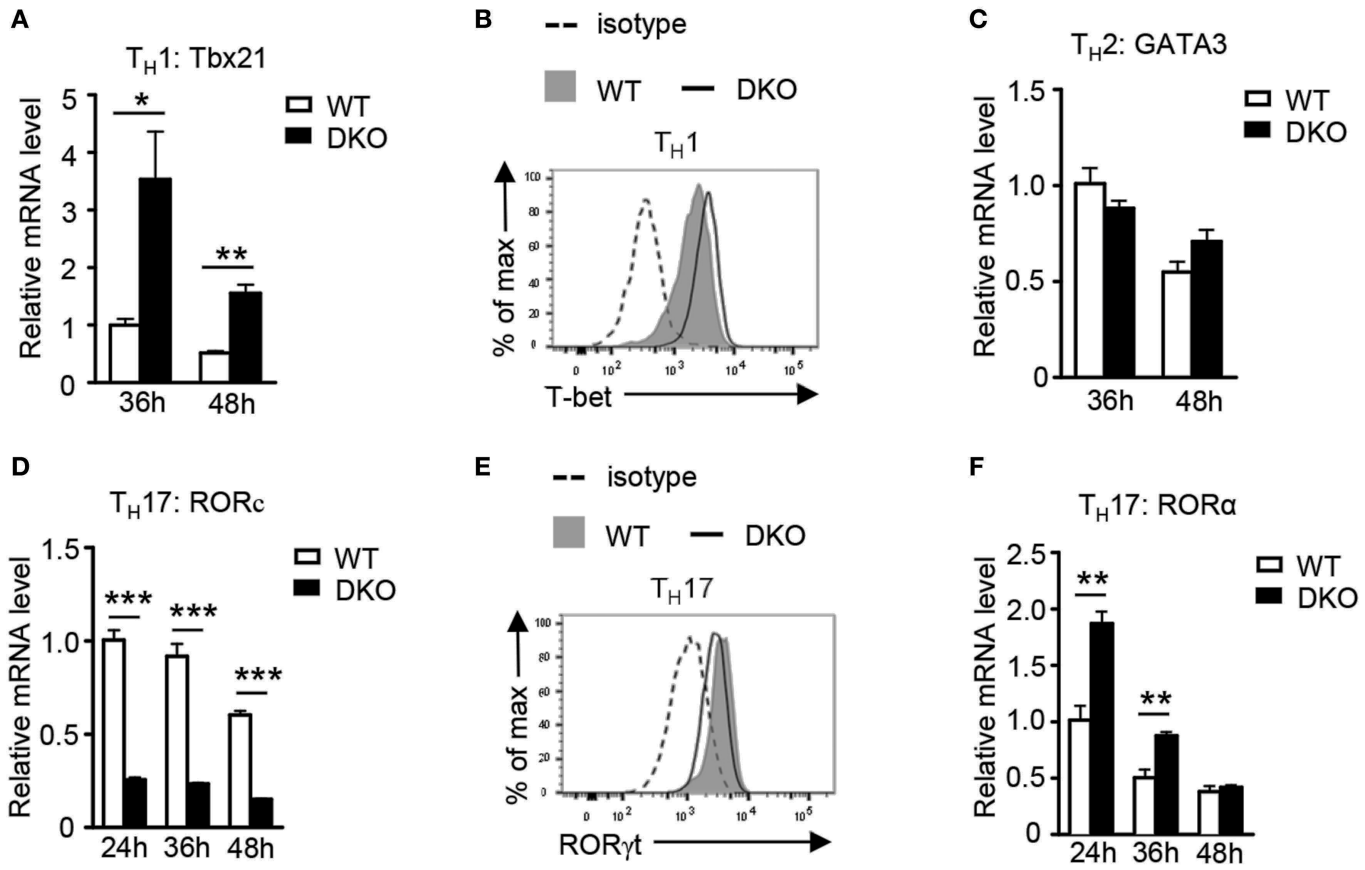
Effects of DGKα and ζ double deficiency on T_H_ lineage-specific transcription factors. **(A,B)** *Tbx21* mRNA **(A)** and T-bet protein **(B)** levels in CD4^+^ T cells during T_H_1 polarization. **(C)** Relative mRNA level of GATA-3 in CD4^+^ T cells during T_H_2 polarization. **(D,E)** *RORc* mRNA **(D)** and RORγt protein **(E)** levels in CD4^+^ T cells during T_H_17 polarization. **(F)** Relative *RORa* mRNA levels in CD4^+^ T cells during T_H_17 polarization. Data shown are representative of five independent experiments. *P < 0.05; **P < 0.01; ***P < 0.001 determined by the Student *t*-test.

T-bet, GATA-3, RORγt, and RORα are transcription factors that play critical roles in T_H_1, T_H_2, and T_H_17 differentiation, respectively. Under the T_H_1 polarization condition, DKO CD4^+^ T cells expressed higher levels of T-bet at both mRNA and protein levels than WT controls (Figures 6A,B), which was consistent with their elevated T_H_1 responses. In contrast, GATA-3 expression in DKO CD4^+^ T cells was not obviously different from WT controls under the T_H_2 polarization condition (Figure 6C), consistent with a minimal effect of DKO on T_H_2 responses as shown in Figure 2. Interestingly, *Rorc* (gene encoding RORγt) mRNA levels were obviously decreased in DKO CD4^+^ T cells under the T_H_17 polarization condition (Figure 6D), although RORγt protein was only slightly decreased (Figure 6E). In contrast, *RORa* mRNA levels were increased in DKO CD4^+^ T cells 24 and 36 h after polarization (Figure 6F). Both RORα and RORγt are important for T_H_17 differentiation and RORγt is considered the master regulator of the Th17 lineage (61–63). It is intriguing that DGKα and ζ double deficiency enhanced Th17 differentiation yet downregulated RORγt expression. Increased RORα expression in DKO CD4^+^ T cells might partially compensate for the decrease of RORγt. Additionally, DGKαζ deficiency might alleviate the requirement of RORγt and promote T_H_17 differentiation via other mechanisms.

### Effects of DGKα and ζ Double Deficiency on mTORC1/S6K1 Signaling During T_**H**_1 and T_**H**_17 Cell Differentiation

**Figure 7:**
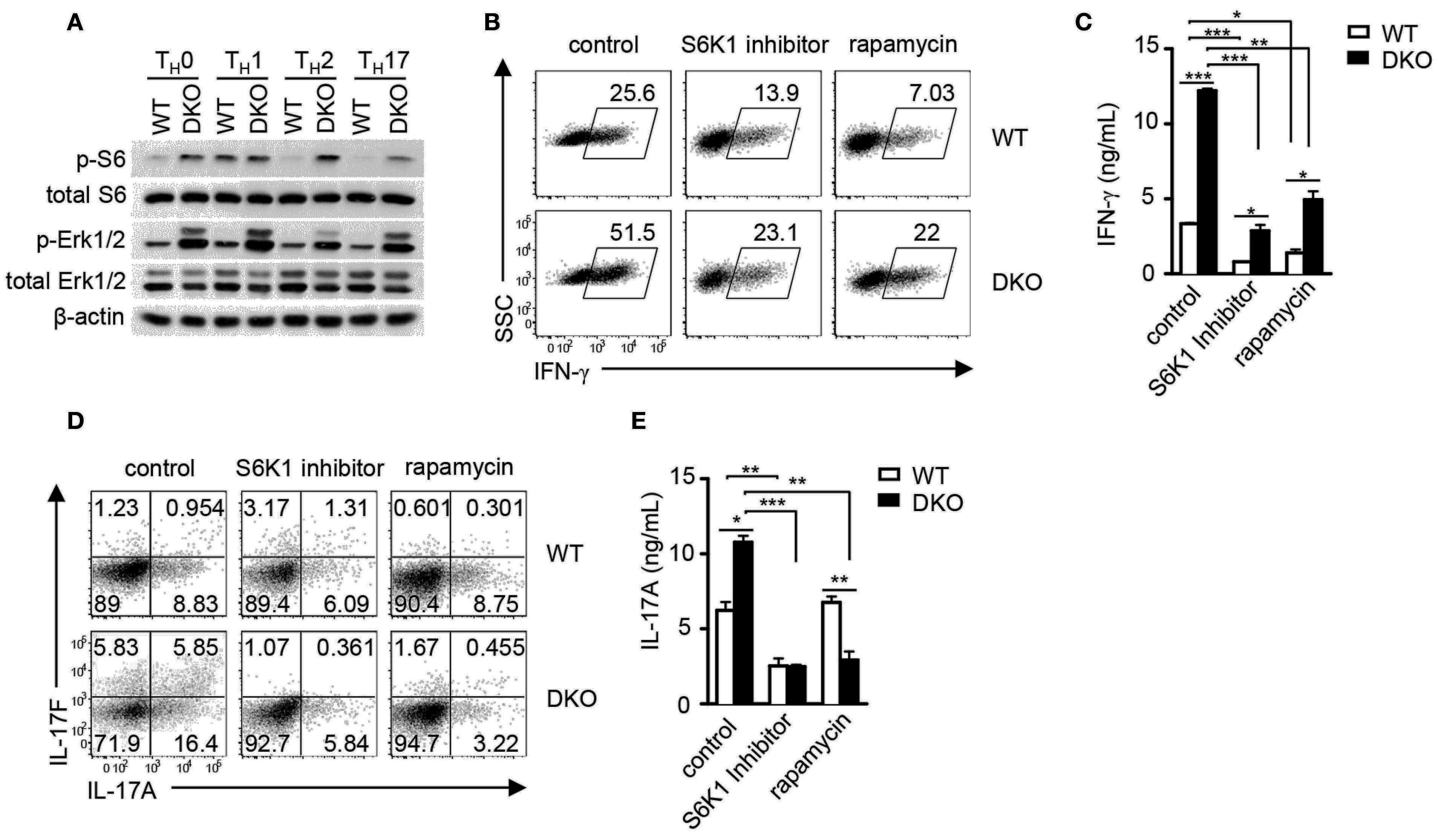
DGKα and ζ negatively regulate mTORC1-S6K1 signaling to control T_H_1 and T_H_17 differentiation. **(A)** Immunoblotting analysis of WT and DKO T_H_ cells after 18-h culture in indicated T_H_ polarization conditions. **(B–D)** Inhibition of T_H_1 and T_H_17 responses *in vitro* by mTORC1/S6K1 inhibition. Naïve WT and DKO CD4^+^ T cells were similarly subjected to *in vitro* T_H_1 and T_H_17 differentiation as described in Figure 1 in the presence or absence of S6K1 inhibitor PF-4708671 (10 μM) and rapamycin (1 nM). **(B)** Representative dot plots of IFN-γ staining on gated CD4^+^ T cells in the T_H_1 polarization condition after PMA and ionomycin stimulation in the presence of GolgiPlug for 4–5 h. **(C)** IFN-γ concentrations in culture supernatants measured by ELISA. **(D)** Representative dot plots of IL-17A and IL-17F staining on gated CD4^+^ T cells under the T_H_17 polarization condition after PMA and ionomycin stimulation in the presence of GolgiPlug for 4–5 h. **(E)** IL-17A concentrations in culture supernatants measured by ELISA. Data shown are representative of two **(A)** and three **(B–E)** independent experiments. *P < 0.05; **P < 0.01; ***P <0.001 as determined by Student *t*-test.

DGKα and ζ negatively control DAG-mediated Ras-Erk1/2 activation in thymocytes and naïve T cells following TCR engagement (36, 38, 54). We further examined how DGKα and ζ double deficiency might affect this pathway during T_H_ polarization. As shown in Figure 7A, Erk1/2 phosphorylation was obviously enhanced in DKO CD4^+^ T cells under T_H_0, T_H_1, T_H_2, and T_H_17 conditions, suggesting that DGKα and ζ negatively controlled Erk1/2 activation during effector CD4^+^ T cell differentiation. Previous studies have found that DAG-mediated RasGRP1-Ras-Erk, PI3K-Akt, and PKCθ-CARMA1 pathways participate in TCR-induced mTORC1 activation and DGKα and ζ double deficiency but not DGKα or ζ single deficiency leads to enhanced mTOR signaling in developing thymocytes (36, 64, 65) and that mTOR plays important roles in Th differentiation (65–69). Although, S6 phosphorylation, an mTORC1/S6K1-dependent event, in T_H_1 cells appeared unaffected by DGKα and ζ double deficiency, it was obviously increased in DKO CD4^+^ T cells under T_H_0, T_H_2, and T_H_17 polarization conditions, suggesting that DGKα and ζ negatively controlled mTORC1 signaling in T_H_0, T_H_2, and T_H_17 cells. Treatment of WT and DKO CD4^+^ T cells with either rapamycin or the S6K1 inhibitor PF-4708671 caused about 50% reduction of IFNγ^+^ cells in both cell types but DKO CD4^+^ T cells still contained higher percentages of IFNγ^+^ cells than WT controls. Thus, DKO CD4^+^ T cells were partially sensitive to mTORC1/S6K1 inhibition (Figures 7B,C), suggesting that additional mechanisms might contribute to enhanced T_H_1 differentiation in these cells. In contrast, T_H_17 differentiation of both DKO and WT CD4^+^ T cells was potently inhibited by either rapamycin or PF-4708671 (Figures 7D,E). Although, we could not rule out potential off-target effects of PF-4708671 and rapamycin, our data suggested that enhanced mTORC1/S6K1 signaling might contribute to the elevated T_H_17 responses of DKO CD4^+^ T cells.

## Discussion

Previous studies have demonstrated that DGKα and ζ play crucial roles in T cell development, activation, anergy, and survival, and CD8 T cell-mediated anti-viral immune responses, *i*NKT cell development, regulatory T cell differentiation, and anti-tumor immune responses (27, 38–54). Additionally, DGKζ has been found to regulate B cell development (70), mast cell activation (71), TLR-mediated innate immunity (72), and NK cells (73). In this study, we have demonstrated that graded DGK activities differentially control CD4^+^ T_H_ differentiation. Although, the absence of either DGKα or ζ selectively impairs T_H_1 differentiation, simultaneous ablation of both DGKα and ζ enhances both T_H_1 and T_H_17 responses *in vitro* and *in vivo*.

Recent studies have demonstrated that mTOR signaling plays a critical role in T cell activation and T_H_ differentiation. mTORC1 promotes T_H_1, T_H_2, and T_H_17 differentiation while mTORC2 activity is indispensable for T_H_2 cells development (65–67). Among different effector CD4^+^ T cells, T_H_1 cells appear to possess the highest S6 phosphorylation and, thus, mTORC1 activity. Although, S6 phosphorylation is not increased in DKO T_H_1 cells, elevated DKO T_H_1 response is substantially decreased when mTORC1-S6K1 signaling is inhibited, suggesting that enhanced DKO T_H_1 response is at least in part via enhanced mTORC1-S6K1 signaling. Different from T_H_1 cells, DKO T_H_0, T_H_2, and T_H_17 cells contain elevated S6 phosphorylation, and inhibition of either mTORC1 or S6K1 reverts their elevated T_H_17 responses. Our study suggested a linkage between DGKs and mTORC1/S6K1 in the regulation of T_H_17 cell differentiation. In thymocytes, T cell line models, and primary T cells, both RasGRP1-Ras-Erk1/2 and PKCθ-CARMA1 pathways signal to promote mTORC1 activation (36, 64). Although, it remains to be defined, DGKα and ζ may inhibit mTORC1/S6K1 signaling via modulating these DAG-mediated signaling pathways during effector CD4^+^ T cell differentiation. In addition to S6K1, many other molecules and pathways that play important roles in T_H_ differentiation are regulated by mTOR (65, 68, 69, 74–77). Future studies should investigate whether DGKα and ζ may regulate T_H_ differentiation through other mechanisms.

Dysregulated T_H_1 and T_H_17 responses contribute to the pathogenesis of numerous autoimmune diseases, including psoriasis, inflammatory bowel disease, rheumatoid arthritis, type 1 diabetes, multiple sclerosis, experimental autoimmune encephalomyelitis, and neutrophil-related airway inflammation (8, 11, 13–15, 18–20). We have shown that dysregulated T_H_1 and T_H_17 responses in the absence of DGKα and ζ are pathogenic, indicated by exacerbated neutrophil-related airway inflammation. Interestingly, DGKα and ζ double deficiency leads to a loss of T cell tolerance and the development of autoimmune diseases in mice (manuscript in preparation). Enhanced CD4^+^ T cell effector function might be an important contributor to the development of autoimmune diseases in these mice. Thus, modulating DGKα and ζ activity could be a potential strategy to shape immune responses. Of note, although DGKα and ζ double deficiency does not obviously affect iTreg induction *in vitro*, our data do not rule out a potential role of DGK activity in peripheral Treg induction from naïve CD4^+^ T cells *in vivo*. Additional studies are needed to determine whether DGKα and ζ play a redundant role in Treg cells.

In summary, DGK activity plays selective roles in T_H_ cell differentiation. A single knockout of DGKα or ζ impaired T_H_1 cell differentiation whereas a deficiency of both DGKα and ζ promoted T_H_1 and T_H_17 cell differentiation *in vitro* and *in vivo*. Such dysregulated expansion of both T_H_ cells in the absence of DGKα and ζ caused severe airway inflammation. DGKα and ζ double deficiency led to enhanced mTORC1-S6K1 activation during T_H_17 cell differentiation, which may contribute to enhanced T_H_17 cell differentiation. Our study demonstrated the role of DGKs in T_H_ cell differentiation and provides useful evidence for these enzymes as potential targets for therapeutic approaches of autoimmune diseases associated with the dysregulation of T_H_1 and T_H_17 cells.

## Data Availability Statement

All datasets generated for this study are included in the article/supplementary material.

## Ethics Statement

The experiments in this study were performed according to a protocol approved by the Institutional Animal Care and Usage Committee of Duke University.

## Author Contributions

JY designed and performed experiments, analyzed data, and wrote the paper. H-XW, JX, LL, and JW performed experiments and analyzed data. EW generated critical reagents. X-PZ conceived the project, designed experiments, participated in data analysis, and wrote the paper.

### Conflict of Interest

The authors declare that the research was conducted in the absence of any commercial or financial relationships that could be construed as a potential conflict of interest.
